# Supplementary material for: A High-Throughput Assay for In Vitro Determination of Release Factor-Dependent Peptide Release from a Pretermination Complex by Fluorescence Anisotropy—Application to Nonsense Suppressor Screening and Mechanistic Studies
Source: Biomolecules. 2023 Jan 27;13(2):242. doi: 10.3390/biom13020242 (PMC9953321; doi:10.3390/biom13020242)
Supplement: Supplementary file 1 [file biomolecules-13-00242-s001.zip › biomolecules-2156558-supplementary.pdf]

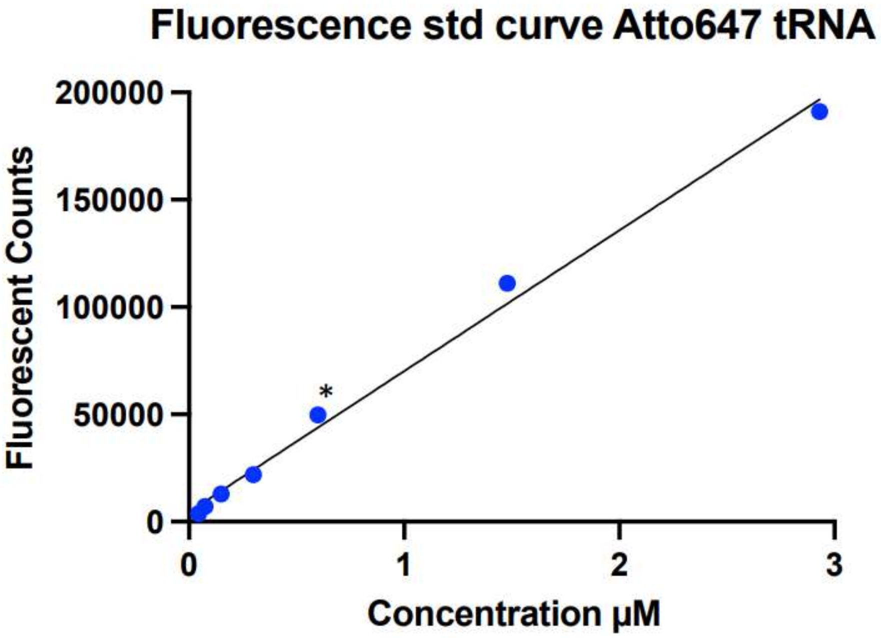

Figure S1 Fluorescence standard curve of Atto647-Lys-tRNA^{Lys}. The asterisk maks the measured value for Atto647-labeled Stop-POST5.

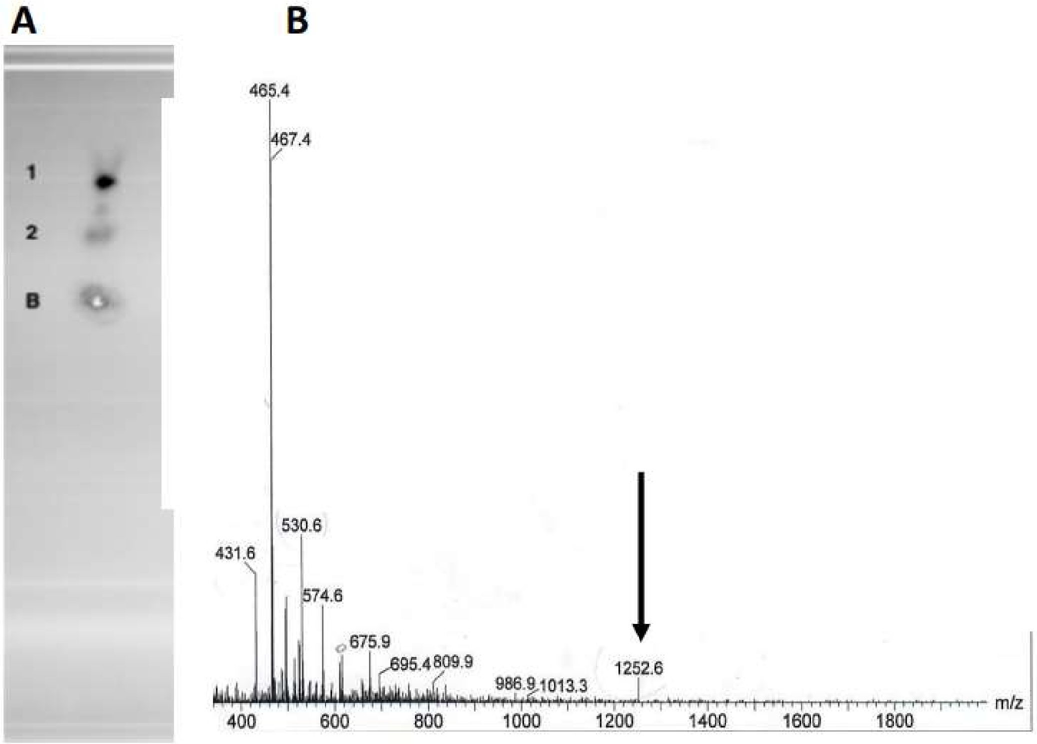

Figure S2. A. Thin layer electrophoresis (TLE) of released FKVRQ Atto647 peptide. 1) FK*^{Atto647}VRQ; 2) Atto647 NHS; B) Baseline. TLE was run on a 20 x 20 cm cellulose plate for 4h in AcOH 110 ml, Pyridine 2.7 ml, H_2O 550 ml, pH 1.7, at 300 Volts. Spots, visualized with a Typhoon™ FLA 7000 reader, were scratched from plate, eluted with MeOH and quantified by [^{3}H]-Glu. **B.** Mass spectrum of FK*^{Atto647}VRQ isolated by TLE from RFC peptide release reactions. Calculated mass: 1252.6
